# Individual vs. combinatorial effect of elevated CO_2 _conditions and salinity stress on *Arabidopsis thaliana *liquid cultures: Comparing the early molecular response using time-series transcriptomic and metabolomic analyses

**DOI:** 10.1186/1752-0509-4-177

**Published:** 2010-12-29

**Authors:** Harin Kanani, Bhaskar Dutta, Maria I Klapa

**Affiliations:** 1 Metabolic Engineering and Systems Biology Laboratory, Department of Chemical & Biomolecular Engineering, University of Maryland, College Park, MD 20742, USA; 2 Metabolic Engineering and Systems Biology Laboratory, Institute of Chemical Engineering and High Temperature Chemical Processes (ICE-HT), Foundation for Research and Technology-Hellas (FORTH), GR-265 04 Patras, Greece; 3 Neogen Chemicals Limited, Thane, MH 400601, India; 4 Biotechnology HPC Software Applications Institute, Telemedicine and Advanced Technology Research Center, U.S. Army Medical Research and Materiel Command, Ft. Detrick, Maryland, USA

## Abstract

****Background**:**

In this study, we investigated the individual and combinatorial effect of elevated CO_2 _conditions and salinity stress on the dynamics of both the transcriptional and metabolic physiology of *Arabidopsis thaliana *liquid hydroponic cultures over the first 30 hours of continuous treatment. Both perturbations are of particular interest in plant and agro-biotechnological applications. Moreover, within the timeframe of this experiment, they are expected to affect plant growth to opposite directions. Thus, a major objective was to investigate whether this expected "divergence" was valid for the individual perturbations and to study how it is manifested under the combined stress at two molecular levels of cellular function, using high-throughput analyses.

****Results**:**

We observed that a) high salinity has stronger effect than elevated CO_2 _at both the transcriptional and metabolic levels, b) the transcriptional responses to the salinity and combined stresses exhibit strong similarity, implying a robust transcriptional machinery acting to the salinity stress independent of the co-occurrence of elevated CO_2_, c) the combinatorial effect of the two perturbations on the metabolic physiology is milder than of the salinity stress alone. Metabolomic analysis suggested that the beneficial role of elevated CO_2 _on salt-stressed plants within the timeframe of this study should be attributed to the provided additional resources; these allow the plants to respond to high salinity without having to forfeit other major metabolic functions, and d) 9 h-12 h and 24 h of treatment coincide with significant changes in the metabolic physiology under any of the investigated stresses. Significant differences between the acute and longer term responses were observed at both molecular levels.

****Conclusions**:**

This study contributes large-scale dynamic omic data from two levels of cellular function for a plant system under various stresses. It provides an additional example of the power of integrated omic analyses for the comprehensive study of the molecular physiology of complex biological systems. Moreover, taking into consideration the particular interest of the two investigated perturbations in plant biotechnology, enhanced understanding of the molecular physiology of the plants under these conditions could lead to the design of novel metabolic engineering strategies to increase the resistance of commercial crops to salinity stress.

## Background

In the recent years, many physiological studies in various organisms concern the elucidation of the molecular mechanisms underlying the response of a particular biological system to combined perturbations [e.g. [1-4]]. These studies are triggered mainly by the fact that in most real-world problems in life sciences in general, and in plant research in particular, the investigated biological systems are under the combinatorial effect of multiple stresses [5]. Moreover, deciphering the relationship between the molecular response of a biological system to individual compared to the combined stresses is of significance for functional genomics [1], molecular genetics [6], metabolic engineering [6,7] and the development of therapeutic and/or other treatments depending on the investigated problem.

In the present study, we investigated the combinatorial effect of elevated CO_2 _conditions and salt (NaCl) stress on both the transcriptional and metabolic physiology of *Arabidopsis thaliana *over the first 30 hours of continuous perturbation. To this end, we employed liquid hydroponic cultures in rich medium, monitoring their "net" (i.e. average over the entire plant) full-genome transcriptional and polar metabolic profiles at various time-points during the treatment period (see [8]). The selection of elevated CO_2 _and salt stress as the target perturbations of this study was based primarily on two reasons: a) both are among the perturbations of particular interest in plant and agro-biotechnological applications [6,9-15], but most importantly because b) individually they are expected to affect plant growth to opposite directions. The salt stress is generally considered negative for the growth of the plants [16,17]; on the other hand, CO_2 _being the main carbon source for the plant cultures, elevated CO_2 _conditions in the growth environment of the plants can be initially beneficial for plant growth [18]. Thus, a major objective of the present study was to investigate the validity of this expected "divergence" for the individual perturbations and study how this is manifested under the combined stress at two molecular levels of cellular function.

"Omic" analyses provide a useful tool for this study as they enable a systemic view of the cellular physiology at each level of cellular function, without requiring extensive knowledge of the structure and regulation of the investigated transcriptional or metabolic networks. The liquid hydroponic culture offers a well-controlled and reproducible experimental framework, which enables the direct comparison of the individual with the combined perturbations excluding interference from other varying parameters. Liquid hydroponic cultures have been used in many high-throughput analyses of plant systems [19-23]. Finally, monitoring the "net" molecular response of the plant cultures enables the observation of global physiological changes characterizing a particular stress, which are unobservable when specific tissues and/or cell types are studied. While missing information about the locally active molecular phenomena, these "holistic" observations could indeed lead to interesting conclusions about the systemic trend of the physiological change [8,19]. The acquired results will be presented in the following sections and discussed in the context of the currently available knowledge about the individual and the combinatorial effect of the elevated CO_2 _conditions and salinity stress on plant physiology.

## Methods

### Experimental Design and Setup

**Figure 1:**
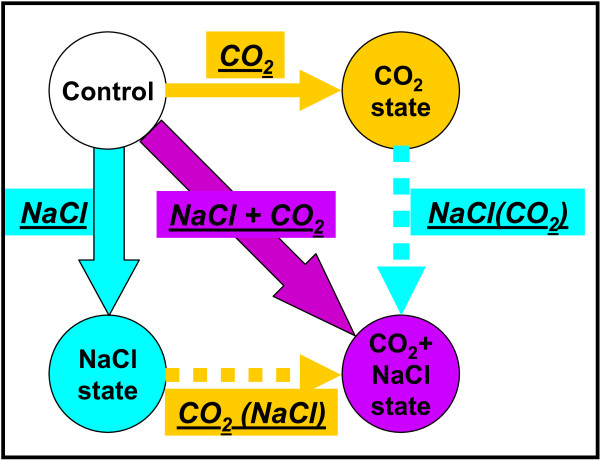
**Simple schematic of the experimental design**. Each arrow depicts the stress that is imposed on the physiological state at the start end of the arrow to reach the physiological state at the end point of the arrow. The nodes of the graph depict the physiological states as they were monitored by the four monitored *A. thaliana *liquid hydroponic culture sets. Based on the particular experimental design, the effect of two perturbations that were not applied experimentally could be implied from the data: 1) the NaCl stress on the under the elevated CO_2 _conditions physiological state (depicted as NaCl(CO_2_) stress), and 2) the elevated CO_2 _conditions on the under the NaCl stress physiological state (depicted as CO_2_(NaCl)).

This study builds on that of the individual elevated CO_2 _perturbation on *A. thaliana *liquid hydroponic cultures presented in [8]. In the present work, the effect of the individual salinity (NaCl) stress and the combined (elevated CO_2 _and NaCl) stress were also investigated. Figure 1 provides a simple schematic of the experimental design including all three perturbations (individual and combined); each arrow corresponds to a perturbation, each node corresponds to a physiological state: i.e. control, CO_2_, NaCl and (NaCl + CO_2_) states. Under a particular perturbation, the plants shift from the physiological state at the starting point to the physiological state at the end point of the corresponding arrow. Data about the control and CO_2 _states were used from [8]. Data about the NaCl and (NaCl + CO_2_) states were generated in the present study from two sets of 20 liquid hydroponic cultures. For their first 12 days, both sets were grown in ambient air under constant white light intensity (80 - 100 μE m^-2 ^s^-2^) and temperature (23°C) inside two different growth chambers (model M-40, EGC Inc., Chagrin Falls, OH). All experimental details regarding seed handling, orbital shaking of the cultures and environmental conditions in the growth chamber during the first 12 days of plant growth are same as in [8]. At the end of the 12^th ^day, 4 liquid hydroponic cultures were harvested from each set to be used as markers of the growth of this set up to that stage. These 4 cultures correspond to the experimental time zero (0 h). Immediately after this harvesting, autoclaved NaCl (purchased from Sigma) solution in de-ionized water was added to all 40 shake-flasks using a syringe, to obtain 50 mM NaCl concentration in the culture medium. At the same time, the CO_2 _concentration in the air of one of the growth chambers was set to 10,000 ppm (see [8] for details). Two cultures were harvested from each set at 1 h, 3 h, 6 h, 9 h, 12 h, 18 h, 24 h and 30 h of the treatment, to monitor the dynamics of the early molecular response of the plants that is triggered by the NaCl and (CO_2_+NaCl) stresses. Each harvested culture was rinsed in distilled water, weighed, frozen in liquid nitrogen and stored at -80°C for further analysis. 3 out of the 40 cultures were excluded from further analysis due to contamination; two at the 0 h and one at the 1 h of the NaCl treatment. The experiments were carried out in the growth chambers of the University of Maryland Green House Facility.

50 mM NaCl was selected as a medium-level salt stress; it has been reported [16] that *A. thaliana *plants can sustain in this stress for four days before severe deteriorating effects becoming apparent. Thus, it was certain that within the timeframe of the present experiment (i.e. 30 hours), any observed physiological changes would reflect the response of the plants to the applied perturbation without any interference from cellular death effects. The more severe stress of 250 mM NaCl, which has also been studied for *A. thaliana *plants [17], causes plant death in two days. On the other hand, the high level of 1% CO_2 _was selected to ensure observable early response from the plants within the timeframe of the present study [8].

### Transcriptional Profiling

Transcriptional profiling was carried out with *A. thaliana *full-genome DNA amplicon microarrays (see [8]). Slide printing, RNA extraction, probe labeling, hybridization, image processing (all carried out in the DNA microarray facilities of the Eukaryotic Genomics laboratory of the Institute for Genomic Research (TIGR) - currently J. Craig Venter Institute -, Rockville, MD 20850, USA) and data normalization were carried out as described in [8]. The data files that were produced after the image processing of the microarrays have been deposited at the public database ArrayExpress under the accession number a) E-MEXP-773 for the control and the CO_2 _states (see [8]) and b) A-MEXP-1593 for the NaCl and (NaCl+CO_2_) states. For each culture set, the expression of a gene at a particular harvest timepoint was estimated as the geometric mean of its expression values in all biological replicates harvested at this time point. If one of the biological replicates was excluded from further analysis, the transcriptional profile of the other was considered as representative of the particular physiological state. Subsequently, the expression of a gene at a particular time point was divided by the expression of the gene at the 0 h time point in the same culture set. These relative to 0 h gene expression profiles over time will be referred to as "control" (or "at the control state"), "under elevated CO_2 _conditions" (or "at the CO_2 _state"), "under the NaCl stress" (or "at the NaCl state") or "under the (NaCl+CO_2_) or combined stress" (or "at the (NaCl+CO_2_) state") in the rest of the text and will be used to extract biologically relevant conclusions. Genes that were detected in less than 75% of the time points for each comparison were removed from further analysis. Any missing expressions were imputed using the k-means neighborhood algorithm [24], as this is implemented in the TM4 MeV (V3.1) software [25].

### Metabolic Profiling

The polar metabolic profiles of the harvested seedlings were acquired as described in [8]. The peak identification and quantification was carried out as described in [26]. The raw metabolomic datasets are provided in a) Supplementary Table 1 of [8] for the control and CO_2 _states, and b) Additional File 1 of this paper for the NaCl and (NaCl+CO_2_) states. Metabolomic profiling data validation, normalization and filtering was carried out based on [26,27], as described in detail in [8]. For each culture set, a metabolite's relative peak area (RPA) at a particular harvest timepoint was estimated as the arithmetic mean of its peak area in all biological replicates harvested at this time point. Subsequently, the RPA of a metabolite at a particular time point was divided by the RPA of this metabolite at the 0 h time point in the same culture set. These relative to 0 h metabolite RPA profiles over time will be referred to as "control" (or "at the control state"), "under elevated CO_2 _conditions" (or "at the CO_2 _state"), "under the NaCl stress" (or "at the NaCl state") or "under the (NaCl+CO_2_) or combined stress" (or "at the (NaCl+CO_2_) state") in the rest of the text and used to extract biologically relevant conclusions. Any missing RPAs were imputed using the k-means neighborhood algorithm [24] as this is implemented in the TM4 MeV (V3.1) software [25].

### Data Analysis

**Figure 2:**
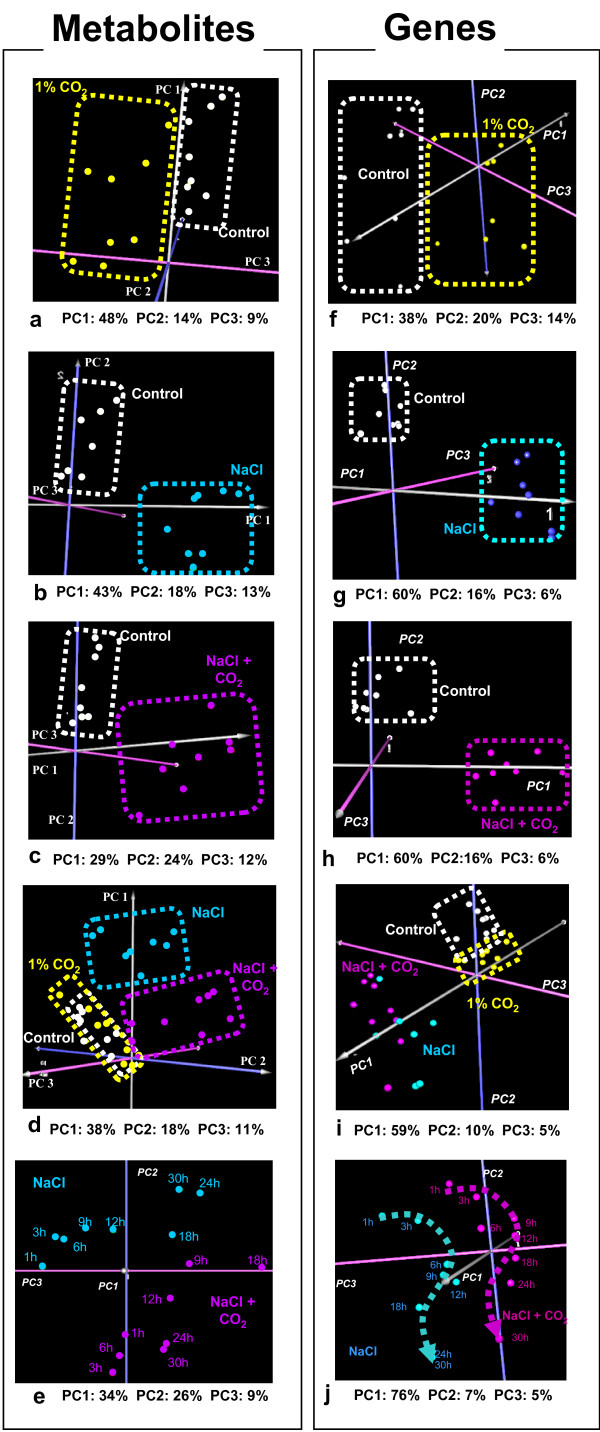
**Principal component analysis (PCA) of the metabolomic (a-e) and transcriptomic (f-j) datasets**. A spot in any of the (a-e) and (f-j) graphs represents, respectively, the metabolic or the transcriptional profile of the plant cultures at one of the experimental timepoints under the stress conditions, the name of which is depicted in the same color.

Principal Component Analysis (PCA) of the metabolomic and transcriptomic profiles was carried out using TM4 MeV (v3.1) software [25] (see Figure 2). The PCA graph enables the visualization of the holistic effect of a perturbation on the transcriptional or metabolic physiology of the plants. Timepoint pair-wise non-parametric significance analysis, using Significance Analysis for Microarrays (SAM) (as implemented in TM4 MeV (v3.1)) and Microarray Time-Series Analysis (MiTimeS) [28], was carried out between two physiological states to identify the genes or metabolites whose expression or concentration, respectively, changed significantly due to the applied perturbation. In all cases, in both transcriptional and metabolic profiling analyses, the threshold of significance (delta) was selected as the minimum corresponding to zero (0) false discovery rate (FDR)-median. Due to the lower number of metabolites compared to genes, in metabolic profiling analysis, MiTimeS was used without the Bonferroni-like correction described in [28]. Additional File 2 includes information about the significance level of all annotated metabolite peaks a) at each time point, as this was identified by MiTimeS, and b) in average over the thirty hours of treatment, as this was identified from paired-SAM, for all five physiological state comparisons indicated in Figure 1.

**Figure 3:**
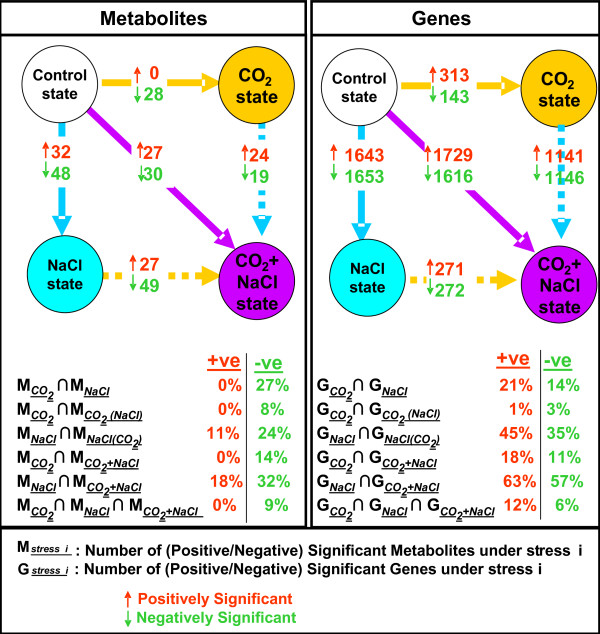
**Number of significant genes and metabolites for any of the investigated perturbations**. A. Number of positively or negatively significant metabolites and genes for any of the applied and implied (as explained in Figure 1) perturbations. B. The number of common (positively or negatively) significant metabolites or genes between two perturbations normalized by the product of the number of (positively or negatively, respectively) significant metabolites or genes for each of the perturbations (%).

**Figure 4:**
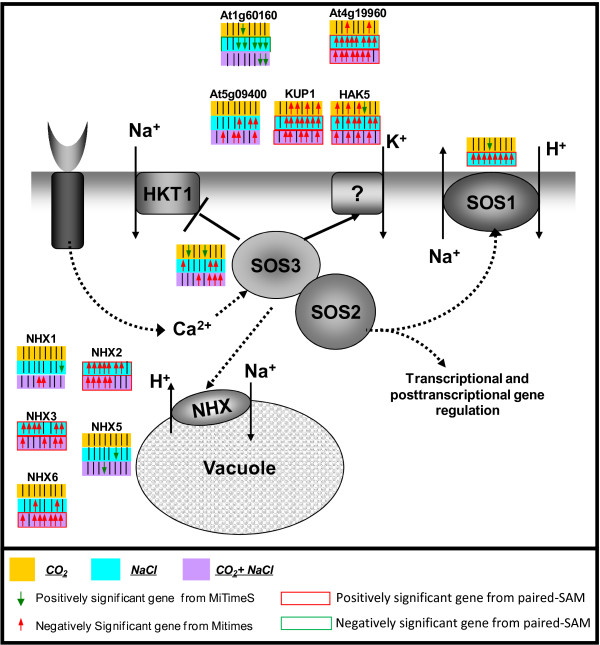
**The significance profile over time and the significance level by paired-SAM of genes in the SOS pathway due to (a) the elevated CO_2 _perturbation (yellow background), (b) the NaCl stress (light blue background) and c) the combined stress (purple background) on the control state**. If a gene was identified as positively, negatively or nonsignificant at the n-th time point for a particular stress, then the n-th short arrow in the box corresponding to the color assigned to the particular stress next to the reaction catalyzed by the protein encoded by the particular gene is upward and colored red, downward and colored green or a black short line, respectively. If the gene was also identified as positively or negatively significant over all time points by paired SAM under the a), b) or c) perturbation, then the frame of the yellow (for a), light blue (for b) or purple (for c) box, is colored red or green, respectively.

**Figure 5:**
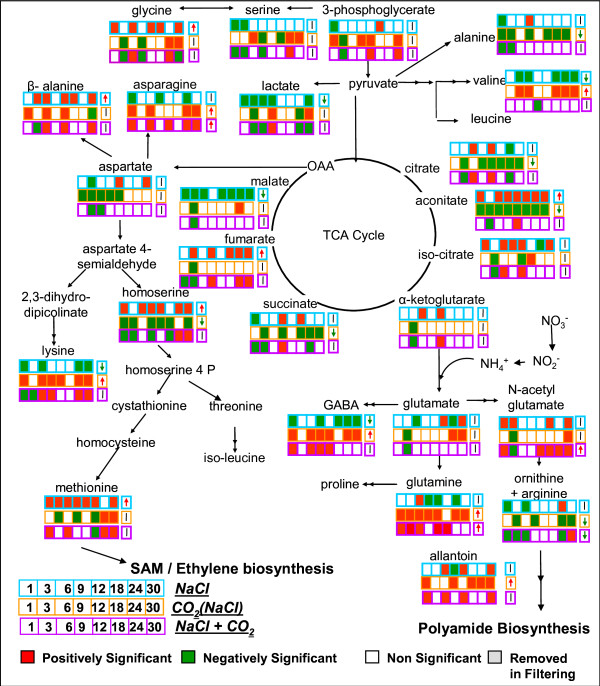
**The significance profile over time and the significance level by paired-SAM of metabolites in the tricarboxylic acid (TCA) cycle and amino acid biosynthesis pathways due to (a) NaCl stress on the control state (blue), (b) the elevated CO_2 _conditions on the NaCl state (orange), and (c) the combined (NaCl + CO_2_) stress on the control state (purple)**. If a metabolite was identified as positively, negatively or nonsignificant at the n-th time point for a particular stress, then the n-th box with the corresponding to the stress borderline color next to the name of this metabolite is colored red, green or remains blank, respectively. The nine-th separate box in each line depicts the significance level of a metabolite for the particular stress as this was identified by paired-SAM. If metabolite was positively, negatively or nonsignificant by paired-SAM, a red upward arrow, a green downward arrow or a black vertical line is shown, respectively, in the nine-th box.

**Figure 6:**
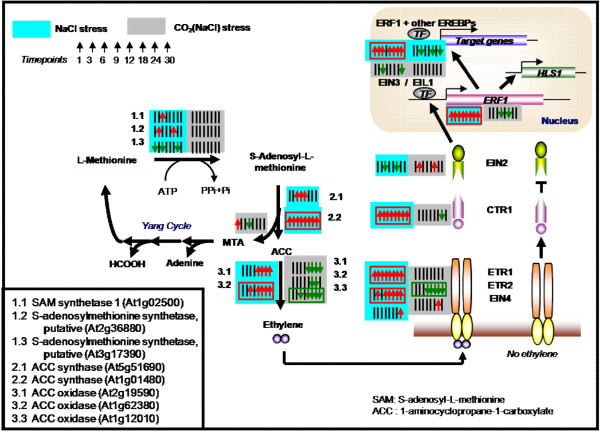
**The significance profile over time and the significance level by paired-SAM of genes in the ethylene biosynthesis and signalling pathways due to (a) NaCl stress on the control state (blue), and (b) the elevated CO_2 _conditions on the NaCl state (gray)**. The convention about the color and the direction of the small arrows is described in the legend of Figure 4.

**Figure 7:**
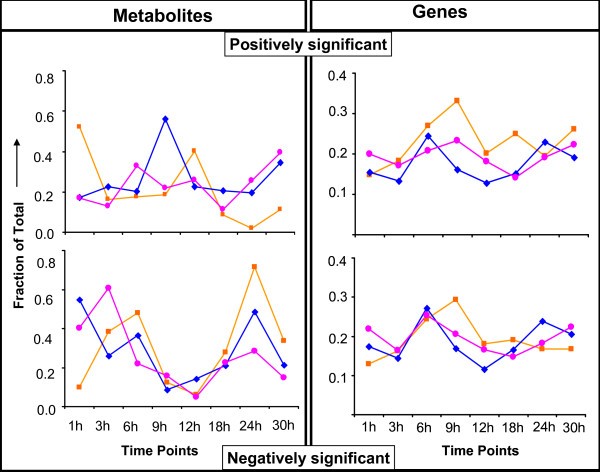
**The time profile of the number of positively or negatively significant metabolites or genes for each of the applied stresses**.

When the lists of significant genes or metabolites have been identified for each physiological state comparison indicated in Figure 1, comparison between the respective lists produced the results shown in Figures 3, 4, 5, 6 and 7 and Tables 1 and 2. Specifically, in Tables 1 and 2 we searched for the metabolites or genes whose concentration or expression, respectively, changed in the opposite direction due to the NaCl stress and the elevated CO_2 _treatment in the presence of the NaCl stress.

**Table 1:** The number of metabolites, which are common between the sets depicted in the row and column of the corresponding element

	Positively significant metabolites in the NaCl compared to the control state [Total number = 32]	Negatively significant metabolites in the NaCl compared to the control state [Total number = 48]
**Positively significant metabolites in the (NaCl + CO_2_) compared to the NaCl state [Total number = 27]**	0	14

**Negatively significant metabolites in the (NaCl + CO_2_) compared to the NaCl state [Total number = 49]**	15	2

**Table 2:** The number of genes, which are common between the sets depicted in the row and column of the corresponding element

	Positively significant genes in the NaCl compared to the control state [Total number = 1643]	Positively significant genes in the NaCl compared to the control state [Total number = 1653]
**Positively significant genes in the (NaCl + CO_2_) compared to the NaCl state [Total number = 271]**	17	66

**Negatively significant genes in the (NaCl + CO_2_) compared to the NaCl state [Total number = 272]**	92	6

## Results and Discussion

High-throughput biomolecular analysis enables the study of the physiological response of a system to a particular perturbation from both the targeted and the holistic perspective. The former focuses on specific pathways and cellular functions that have been related to or are of special interest with respect to the particular physiological conditions. The latter exploits the systemic view of the physiological state that enables the correlation between parallel-occurring phenomena towards the elucidation of biomolecular mechanisms that underlie a particular physiological response. In this way, the holistic analysis could reveal pathways or functions that have not until now been related to the investigated physiological conditions based on the currently available knowledge. We combined information from both perspectives to study a) the difference in the net physiological response of the plants between the various perturbations at either the transcriptional or the metabolic levels, and b) the difference between the transcriptional and metabolic response of the plants for each perturbation. In the following paragraphs, the major observations about these comparisons are discussed.

### Observation 1

*The net holistic effect of the salinity stress on the hydroponic culture physiology is stronger than that of the elevated CO_2 _conditions at both the transcriptional and metabolic levels during the first 30 hours of treatment*

This observation, which is more evident at the metabolic level, is supported by the PCA results shown in Figures 2a, f and 2b, g. The metabolic profiles of the plants subjected to the elevated CO_2 _conditions compared to those of the control plants differ on PC3, which carries only 9% of the data variance in the original datasets (Figure 2a). On the other hand, the metabolic profiles of the plants subjected to the salinity stress differ clearly on PC1 and PC2 with respect to their control counterparts (see Figure 2b). PC1 and PC2 carry 61% of the variance in the original datasets. At the transcriptional level, the effect of the elevated CO_2 _conditions is stronger than at the metabolic level (Figure 2f). However, the transcriptional effect of the NaCl stress is clearly more prominent (Figure 2g) on both PC1 (60% of variance) and PC2 (16% of variance). The stronger effect of the NaCl stress compared to the elevated CO_2 _conditions is also clear by the number of the metabolites or genes whose concentration or expression, respectively, changes significantly between the control and the perturbed states (see top graphs in Figure 3).

The stronger effect of the medium-level salinity stress over the elevated CO_2 _conditions is observed despite the fact that the imposed CO_2 _increase in the growth environment of the plant cultures is very high compared to CO_2 _concentration in the ambient air. The mild early effect of the elevated CO_2 _conditions could be attributed to the fact that, while normally growing, the plants do not require and consequently do not use the additional resources provided from the CO_2 _excess. Thus, their physiological state under the elevated CO_2 _conditions does not greatly differ from the control. On the other hand, the presence of elevated salinity in the growth environment of the plants triggers defense mechanisms right away, affecting thus the physiological state of the plant cultures from early on.

### Observation 2

Strong similarity was observed between the transcriptional responses of the hydroponic cultures to the NaCl and (NaCl + CO_2_) stresses

This observation is supported from the PCA graphs g-h in Figure 2. The similarity is validated from the PCA graph i in the same figure, which includes all timepoint transcriptional profiles in all four physiological (i.e. control, CO_2_, NaCl and (NaCl+CO_2_)) states. Interestingly, zooming in the 2i PCA graph (see Figure 2j) shows that the NaCl and (NaCl+CO_2_) transcriptional profiles follow also the same trend with time. This observation indicates similarity even in the dynamics of the early transcriptional response of the plant cultures to the two stresses. The similarity suggests that the early transcriptional response of the plant cultures to the salinity stress is robustly active independently of the co-occurrence of the elevated CO_2 _conditions. This similarity is further validated from the number of positively and negatively significant genes that are common between the two stresses, compared to the total number of positively and negatively, respectively, significant genes that are associated with each stress (see lower part of Figure 3 and Additional File 3). The normalized intersection percentage is 63% and 57%, respectively, for the positively and negatively significant genes (Figure 3). These are the highest intersection percentages between any pair (or triplet) of the investigated perturbations. The list of the negatively significant genes that are common between the salinity and the combined stresses includes mainly genes encoding for proteins that are involved in the cellular growth and maintenance, like histone subunits, ribosomal small and large subunits, xyloglucan transferase, photosystems I and II subunit and RuBisCO subunit binding proteins. On the other hand, the common between the two stresses positively significant genes include a large number of protein kinase genes in general and inositol and MAPK signaling genes in particular, underscoring the importance of the signaling reactions under the salinity stress. The complete gene list of the common between the two stresses positively and negatively significant genes is provided in Additional File 4.

Figure 4 shows the transcriptional response of the SOS pathway to the elevated CO_2 _conditions compared to the salt and combined stresses. The activity of the SOS signaling pathway is of particular interest regarding the response of the plants to salt stress [29]. This pathway is responsible for the extracellular and vacuolar sequestration of the Na^+ ^ions, a process of high significance for the ion homeostasis of the plants. The Na^+ ^ion increase caused by the salt stress could be detrimental to the plants, causing membrane disorganization, impaired nutrient and water acquisition, metabolic toxicity, inhibition of photosynthesis, and production of reactive oxygen species [30]. As validated from Figure 4, the activity of the SOS pathway was significantly increased at the transcriptional level under the salinity and the combined stress conditions. This observation is in agreement with our holistic suggestion that the early transcriptional response of the plant cultures to the salinity stress is robustly active independently of the co-occurrence of the elevated CO_2 _conditions.

### Observation 3

The combinatorial effect of the elevated CO_2 _conditions and salinity stress on the metabolic physiology of the plants is milder than that of the salinity stress alone

While the transcriptional responses of the plants to the salinity and the combined stresses were similar, this appeared not to be the case for the metabolic responses. Observation 3 becomes apparent from the PCA graph of the metabolic profiles from all stresses (Figure 2d). The metabolic profiles of the NaCl state are clearly of different coordinates on principal component 1 (accounting for 38% of the data variance in the original experimental space) than the control metabolic profiles. However, the metabolic profiles of the (NaCl + CO_2_) state are of similar to the control coordinates on principal component 1, differentiating mainly on the plane of principal components 2 and 3. Viewed from the perspective of the systemic analysis, this observation provides an example of "inconsistency" between the transcriptional and the metabolic levels; these "inconsistencies" support the need for integrated analyses to achieve comprehensive understanding of the molecular physiology. Moreover, the observed metabolic difference between the salinity and the combined stresses carries biological significance about the role of the elevated CO_2 _in alleviating the metabolic effect of the salinity stress on the plant cultures when these two perturbations are combined.

According to our observations, the combined application of the elevated CO_2 _conditions and the salinity stress appears to be beneficial to the plants at the metabolic level compared to the effect of the salinity stress alone, at least for the first 30 hours of treatment that are examined in this study. It is known that to counteract the NaCl stress, the plants are in need to redirect their metabolism towards the production of osmoprotectants [6,31] and antioxidants [32], using part of their available carbon and energy resources for appropriate flux redistribution. Increase of the CO_2 _concentration in the growth environment of the plants provides additional resources. Thus, the elevated CO_2 _conditions could indeed help alleviate part of the negative effect of the salinity stress on the metabolic state of the plants. With the additional resources under the combined perturbation, the plants are expected to be able to send flux towards the production of osmoprotectants and antioxidants, without having to forfeit most of the processes that characterize the control metabolic state.

Data shown in Figure 5 are consistent with this hypothesis. Figure 5 depicts the tricarboxylic acid (TCA) cycle and part of the amino acid biosynthesis pathways. The state comparisons shown are a) the NaCl with respect to the control state, b) the (NaCl + CO_2_) with respect to the NaCl state, indicating indirectly the effect of the elevated CO_2 _conditions on the NaCl-stressed plants, and c) the (NaCl + CO_2_) with respect to the control state. The pathway network is appropriately color-coded; when the change in the (average over the entire plant) concentration of a metabolic intermediate between two metabolic states was identified as positively, negatively or non- significant at a timepoint by MiTimeS and/or in average over all time points by paired-SAM, then the corresponding box is shown as red, green or blank, respectively. Specifically, the NaCl state was characterized by the significant increase in the concentration of homoserine, methionine and β-alanine at most of the timepoints; they were also identified as positively significant from paired-SAM. Moreover, glycine, N-acetylglutamate and allantoin were identified with significantly increased concentration at five, four and three timepoints, respectively. Furthermore, the concentration of all TCA cycle intermediates from citrate to fumarate increased as a consequence of the applied salt stress. All these observations are in accordance with the need of the plants to produce osmoprotectants and antioxidants to counteract the stress conditions. Homoserine and methionine are precursors of the S-Adenosyl-Methionine (SAM) and the ethylene precursor, 1-amino-cyclopropane-1-carboxylic acid. SAM is the most important methyl donor in plants; it is required along with glycine for the biosynthesis of glycine-betaine, which is the main osmoprotectant in *A. thaliana*, and along with β-alanine for the production of β-alanine betaine. There is currently only one study suggesting an osmoprotecting role for β-alanine betaine in the plant family Plumbaginaceae [33]. Both the SAM methylation activity [34,35] and the ethylene production [36] have been shown to increase in plants in response to the salt stress. N-acetyl-glutamate and allantoin are precursors of polyamines. It has been shown that increased polyamine levels in trees and a number of other plant species impart osmoprotection [37,38]. In addition to these observations, the data provided in Additional File 2 indicated that all known fatty acids and sterols, including tocopherol, that were measured in the metabolic profiles exhibited a significant increase in their concentration during the first 12 hours of salt stress. The concentration of sterols was identified as significantly increased under the NaCl stress at 24 h and 30 h of treatment too, and from overall SAM analysis. These observations suggest an important role of sterols in the response of the *A. thaliana *to the salt stress, which has not to-date been thoroughly studied. Currently, only tocopherol has been reported to be produced as anti-oxidant due to the salt stress and it is the first time that other sterols and fatty acid pools are implicated in the response of the plants to this stress, providing the basis for further research. Finally, it is important to note that the significantly increased production of the amino acids/amine-group containing metabolites - precursors of osmopotectants and anti-oxidants discussed above is accompanied by a significant decrease in the products of the competing pathways, i.e. lysine concentration decreased while that of homoserine and methionine increased; asparagine concentration decreased while that of β-alanine increased; and 4-aminobutyric acid (GABA) concentration decreased while that of N-acetylglutamate and allantoin increased. These observations suggest that in light of the available carbon and energy resources, to counteract the salt stress the plants have to forfeit certain functions towards the production of osmoprotectants and antioxidants.

On the other hand, Figure 5 also shows that for many of the depicted metabolites (e.g. intermediates of the "right-hand" side of the TCA cycle, alanine, valine, lysine, asparagine), the significance profile over time due to the NaCl stress on the control state is opposite to the significance profile over time due to the elevated CO_2 _conditions on the NaCl metabolic state. This indicates that the significantly increased or decreased (with respect to the control) concentration of these metabolites at the NaCl metabolic state decreased or increased, respectively, due to the elevated CO_2 _conditions on the NaCl metabolic state. Over the entire set of the measured metabolites, Table 1 indeed shows that a large number of the metabolites, the change in the concentration of which between the (NaCl+CO_2_) and the NaCl state was identified as positively or negatively significant from paired-SAM, were identified in the opposite significance level (i.e. negatively or positively significant, respectively) between the NaCl and the control state (see complete list in Additional File 5); Table 2 shows the corresponding results for the significant genes (the list of the 92 + 66 genes in the diagonal elements is shown in Additional File 5). Thus, the concentration of these metabolites at the (NaCl + CO_2_) state approaches their value at the control conditions. In the same context, many of the metabolites, which were identified from paired-SAM analysis as significantly changing in concentration from the control to the NaCl state (e.g. aconitate, valine, lysine, beta-alanine, lactate, GABA in Figure 5) were identified as non-significant in the comparison between the (NaCl+CO_2_) and the control states. All annotated metabolites with this characteristic can be seen in Additional File 2. However, the concentrations of glycine and methionine, which are related to the synthesis of the osmoprotectant glycine-betaine, remain positively significant at most time points under the combined stress. The same was true for the N-acetyl-glutamate and allantoin, which are precursors of polyamines. These results indicate that the production of osmoprotectants is active at the (NaCl + CO_2_) state, while the concentration of many metabolites does not change significantly from the control state. These observations are consistent with our previously stated hypothesis, based on which, under the combined stress, the plant cultures have enough resources to produce the required osmoprotectants without having to significantly reshuffle the sizes of most metabolite pools to appropriately reorganize their flux distribution. Alleviation of the effect that the salinity stress has on the plants in the presence of the elevated CO_2 _was also observed in the context of the ethylene signaling pathway based on the transcriptomic data (Figure 6). Under the NaCl stress, the expression of most of the ethylene signaling genes was significantly increased with respect to the control state. The comparison of the (NaCl+CO_2_) state with respect to the NaCl state indicated a decrease in the expression of these genes under the combined stress.

The observed beneficial effect of the elevated CO_2 _on the metabolic response of the plant cultures to the salinity stress is in agreement with a recent study on two barley cultivars that reported mitigation of the oxidative stress induced by increased salinity through the application of elevated CO_2 _[5]. In [5], the beneficial role of elevated CO_2 _was attributed to enhanced maintenance of the redox potential due to an elevated rate of CO_2 _assimilation and lower photorespiration. Previous studies in salt-stressed alfalfa [39], pine [40] and oak [41] also report the protective role of elevated CO_2_. Further studies in which elevated CO_2 _conditions are applied to NaCl stressed plants are required to investigate the general validity and the extent of our discussed hypothesis about the mechanisms underlying the enhanced metabolic response of the plant cultures to the (NaCl+CO_2_) stress. Enhanced understanding of these mechanisms could lead to the design of novel metabolic engineering strategies to increase resistance of commercial crops to the salinity stress.

### Observation 4

Differences in the dynamics of the transcriptional and metabolic responses between the various stresses

Studying the graph depicting the number of positively and negatively significant metabolites or genes at each time point (Figure 7), the following observations can be made:

a) Elevated CO_2 _conditions invoke the highest number of positively and the lowest number of negatively significant metabolites during the first hour of treatment, while the opposite (i.e. lowest number of positively and highest of negatively significant) was observed for the NaCl and the (NaCl+CO_2_) stresses.

b) The results from all stresses indicate 9 h-12 h of treatment as coinciding with a significant change in the metabolic physiology of the plants for any of the investigated stresses; for the NaCl stress the lowest number of negatively and the highest number of positively significant polar metabolites was observed at 9 h, while for the elevated CO_2 _conditions at 12 h of treatment; for the combined stress the lowest number of negatively significant metabolites was observed at 12 h of treatment too, however no significant changes in the number of positively significant metabolites were observed throughout the course of the treatment.

c) The time profile of the number of negatively significant metabolites for all stresses indicates 24 h of treatment as coinciding with significant changes in the metabolic physiology of the plants. This is more apparent for the plants under elevated CO_2 _conditions, for which the very high number of negatively significant metabolites at 24 h of treatment is combined with a very low number of positively significant. In [8], 24 h of elevated CO_2 _was indicated as potentially the time at which the plants close their stomata and the rate of carbon fixation and photosynthesis becomes significantly low. The NaCl stressed plants show a lower than the plants under elevated CO_2 _conditions number of negatively significant metabolites at the same time point. This number is similar to the number of negatively significant metabolites for NaCl stressed plants at the first 3 h of the treatment. The number of negatively significant metabolites at 24 h due to the combined stress is the lowest among the three stresses, indicating potentially less stressful conditions for the metabolism of the plants under this perturbation;

d) On the other hand, no major changes in the number of positively or negatively significant genes were observed throughout the treatment period for any perturbation or among the three perturbations. Without being as prominent as in the case of metabolic profiles (see observations b and c above), 6 h-9 h and, in the case of the NaCl stress also 24 h, of treatment correspond to maxima of the time profiles of the number of positively and negatively significant genes. Moreover, in the timepoint correlation networks that are constructed based on the number of the common positively or negatively significantly genes between two time points [see [28]], time points 1 h and 30 h are not connected for any of the investigated stresses (Additional File 6). This indicates a significant change in the transcriptional physiology of the plants after 30 h of treatment. It is also interesting to observe that the 1 h and 3 h time points are connected in 10 out of the 12 correlation networks shown in Additional File 6 for both metabolites and genes and so do the 24 h and 30 h time points. These observations indicate difference between the acute and the longer-term responses of the plants to the applied stresses during the first 30 h of treatment. These results support the selected experimental design regarding the treatment duration and harvest time points studied in this work.

## Conclusions

In the present study, we investigated the combinatorial effect of elevated CO_2 _conditions and salt (NaCl) stress on both the transcriptional and metabolic physiology of *A. thaliana *hydroponic cultures over the first 30 hours of continuous perturbation. The experimental design enabled us to compare the dynamics of the early transcriptional and metabolic responses of the plants between the various perturbations and also compare the transcriptional with the metabolic response for each perturbation. We observed that a) the net holistic effect of the salinity stress was stronger than that of the elevated CO_2 _conditions at both the transcriptional and metabolic levels, b) there was a strong similarity between the transcriptional response of the cultures to the salinity and the combined stress, but c) the combinatorial effect of the elevated CO_2 _conditions and salinity stress on the metabolic physiology of the plants was milder than that of the salinity stress alone and d) there were differences in the acute and the longer-term responses of the plants to any of the stresses during the first 30 h of treatment. At the scientific level, the particular study contributes large-scale dynamic omic data from two levels of cellular function for a plant system under various stresses, and provides an additional example of the power of integrated omic analyses for the comprehensive study of the molecular physiology of complex biological systems. Moreover, taking into consideration the particular interest of the two investigated perturbations in plant and agro-biotechnological applications, enhanced understanding of the molecular physiology of the plants under these conditions could lead to the design of novel metabolic engineering strategies to increase resistance of commercial crops to the salinity stress.

## Authors' contributions

HK participated in the design of the study, carried out along with BD the plant growth experiments, performed both the experimental and computational parts of the metabolomic analysis, collaborated with BD and MIK in the integrated analysis of the transciptomic and metabolomic data, and helped to draft the manuscript. BD participated in the design of the study, carried out along with HK the plant growth experiments, carried out both the experimental and computational parts of the transcriptomic analysis, collaborated with HK and MIK in the integrated analysis of the transciptomic and metabolomic data, and helped to draft the manuscript. MIK conceived the study, participated in its design, coordinated the experiments and the statistical analysis, helped to draft and finalized the manuscript. All authors read and approved the final manuscript.

## Acknowledgements

We would like to gratefully acknowledge the financial support of a) US NSF (QSB-0331312) and b) the UMD Chemical and Biomolecular Engineering Department that funded the PhD fellowships of HK and BD and all expenses related to the experiments of this study; c) FORTH/ICE-HT for funding the writing and publication expenses of this study. We would also like to thank a) Dr. J. Quackenbush for providing access to the Eukaryotic Genomics laboratory of TIGR and its DNA microarray facilities and for his assistance with the DNA microarray measurements and b) the UMD Green House facility for providing access to the growth chambers.

## Supplementary Material

Additional file 1 **Raw Metabolomic Data for the NaCl and (NaCl+CO_2_) states**. This file contains the raw peak areas for the metabolites of known chemical category from the metabolic profiles of the NaCl and (NaCl+CO_2_) states. Click here for file

Additional file 2 **Significance level of all annotated metabolites for all investigated perturbations**. This file indicated the significance level of all annotated metabolites included in the analyses at each individual time point (as estimated by MiTimeS) and in average over the duration of the treatment (as estimated by SAM) for all investigated perturbations. Click here for file

Additional file 3 **Number of common positively and negatively significant genes and metabolites between the elevated CO_2 _conditions, the NaCl and the combined stresses, as identified from paired-SAM**. Venn diagrams of the positively and negatively significant genes and metabolites in the elevated CO_2 _conditions, the NaCl and the combined stresses. Click here for file

Additional file 4 **Common significant genes between the salinity and the combined stresses**. The list of the 1060 and 932 common positively and negatively, respectively, significant genes between the salinity and the combined stresses. The significant genes were identified from paired SAM analysis. Click here for file

Additional file 5 **The list of significant genes and metabolites being at the opposite significance level under the NaCl and CO_2_(NaCl) stresses**. The list of significant genes and metabolites being at the opposite significance level under the NaCl and CO_2_(NaCl) stresses; their number is indicated in the Tables 1 and 2 and discussed in the relevant text. Click here for file

Additional file 6 **The Significance Correlation Matrix (SCM) networks for the positively and negatively significant metabolites and genes for the elevated CO2 conditions, the NaCl and the combined stresses**. The SCM networks for the significant metabolites are shown in figures (a - f) and for the genes in figures (g-l). The construction of the SCM networks is described in detail in [29]. Click here for file
