# Supplementary material for: Individual vs. combinatorial effect of elevated CO2 conditions and salinity stress on Arabidopsis thaliana liquid cultures: Comparing the early molecular response using time-series transcriptomic and metabolomic analyses
Source: BMC Syst Biol. 2010 Dec 29;4:177. doi: 10.1186/1752-0509-4-177 (PMC3027597; doi:10.1186/1752-0509-4-177)
Supplement: Additional file 3 — Number of common positively and negatively significant genes and metabolites between the elevated CO2 conditions, the NaCl and the combined stresses, as identified from paired-SAM. Venn diagrams of the positively and negatively significant genes and metabolites in the elevated CO2 conditions, the NaCl and the combined stresses. [file 1752-0509-4-177-S3.PDF]

Additional File 3

Number of common positively and negatively significant genes and metabolites between the elevated CO_2, the NaCl and the combined stresses, as identified from paired-SAM.

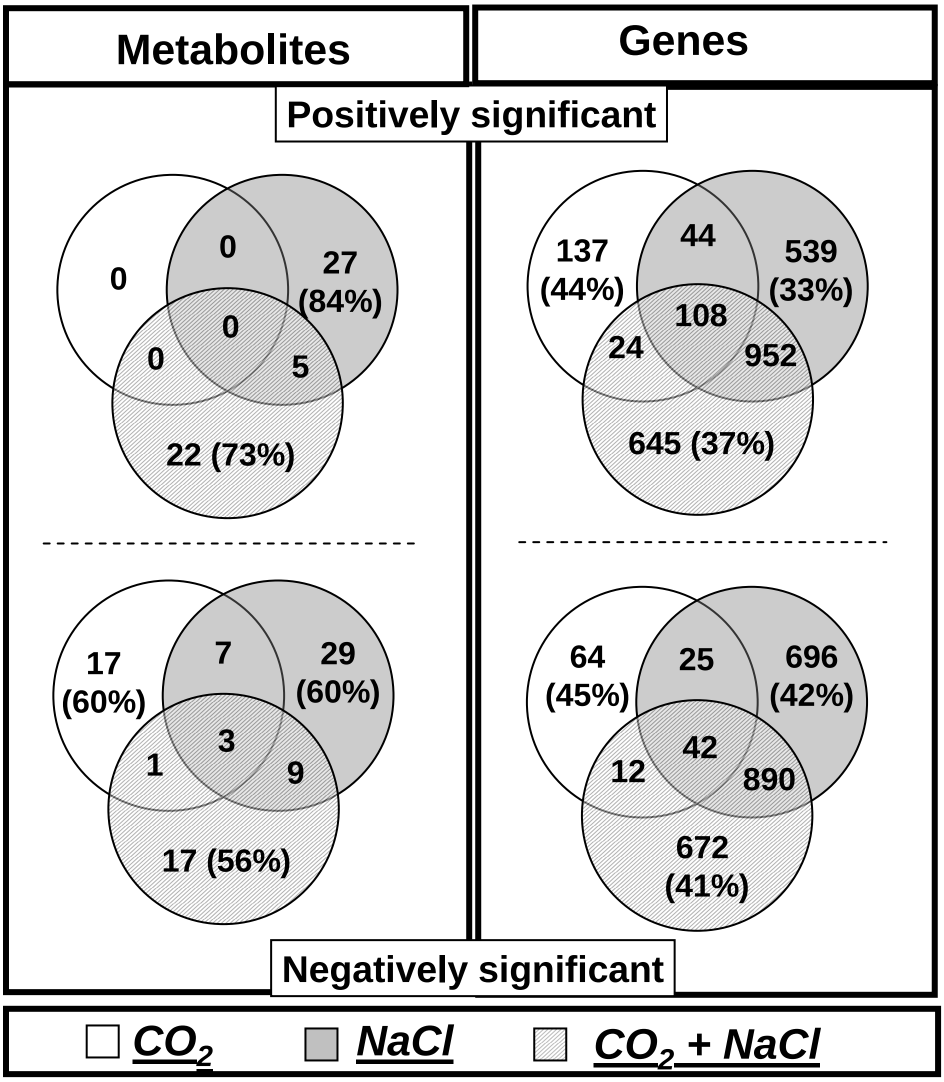

The percentage in parenthesis indicates the fraction of the genes or metabolites that were identified as positively or negatively significant only under the particular stress in the total number of genes or metabolites that were used in the analysis of the particular stress.
