# Supplementary material for: Individual vs. combinatorial effect of elevated CO2 conditions and salinity stress on Arabidopsis thaliana liquid cultures: Comparing the early molecular response using time-series transcriptomic and metabolomic analyses
Source: BMC Syst Biol. 2010 Dec 29;4:177. doi: 10.1186/1752-0509-4-177 (PMC3027597; doi:10.1186/1752-0509-4-177)
Supplement: Additional file 6 — The Significance Correlation Matrix (SCM) networks for the positively and negatively significant metabolites and genes for the elevated CO2 conditions, the NaCl and the combined stresses. The SCM networks for the significant metabolites are shown in figures (a - f) and for the genes in figures (g-l). The construction of the SCM networks is described in detail in [29]. [file 1752-0509-4-177-S6.PDF]

Additional File 6

Significance Correlation Matrix (SCM) Networks for positively and negatively significant metabolites (a to f) and genes (g-l) for the elevated CO_2, NaCl and combined stresses.

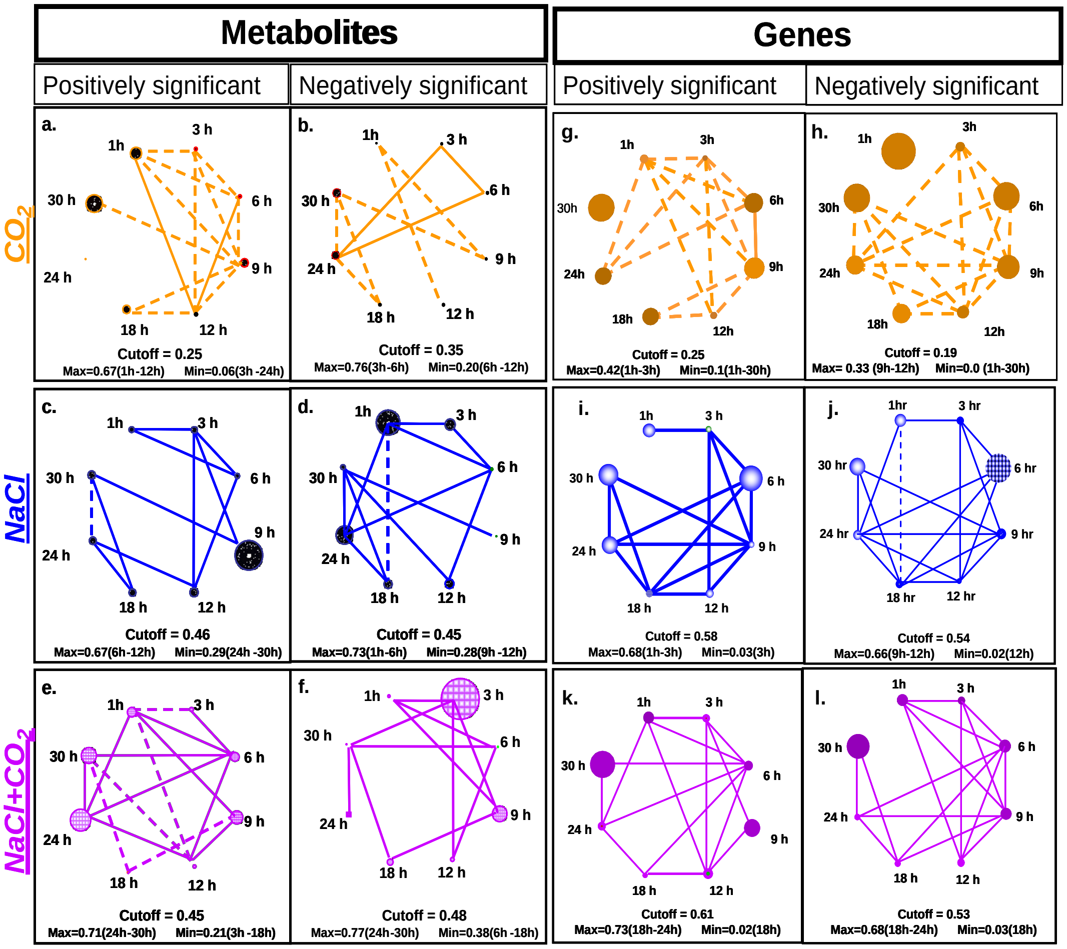

The construction of the SCM networks is described in detail in [28]
